# Supplementary material for: Effectiveness of percutaneous key lesion endoscopic lumbar decompression for the treatment of lumbar spinal stenosis in octogenarian patients
Source: PLoS One. 2024 Nov 21;19(11):e0300836. doi: 10.1371/journal.pone.0300836 (PMC11581282; doi:10.1371/journal.pone.0300836)
Supplement: Attachment — Submitted filename: Response to Reviwers.docx [file pone.0300836.s001.docx]

Tadashi Ito, PhD

Academic Editor, *PLOS ONE*

Dear Dr. Ito,

We appreciate the opportunity to revise our manuscript entitled, “Effectiveness of percutaneous key lesion endoscopic lumbar decompression for the treatment of lumbar spinal stenosis in octogenarian patients.” The manuscript ID is PONE-D-23-21132, authored by Chien-Tung Yang et al. Our responses to the reviewer’s comments are included in the next page.

We have, following the suggestions from reviewers, made every effort to observe long-term changes in imaging after surgery. Despite challenges in extending follow-up due to age-related issues, we have still obtained data from a subset of patients and incorporated it into the revised version. Additionally, we have removed the funding information that originally appeared in the acknowledgment section to comply with PLOS ONE guidelines. The current funding statement is as follows: “CDC was supported by China Medical University Hospital [DMR-110-217] for providing technical and financial support (<https://www.cmuh.cmu.edu.tw/Home/CmuhIndex_EN>). The funders had no role in the study design, data collection, and analysis, decision to publish, or preparation of the manuscript, and remains accurate and unchanged.”

To comply with PLOS ONE’s publication criteria regarding data availability, we have submitted all original X-ray and MR images used to measure spinal parameters. Additionally, we have amended the statement as follows: “For spinal MR and X-ray images employed in the measurement of image parameters, the image files can be accessed on figshare (DOI: 10.6084/m9.figshare.25051325).”

Your kind consideration of our manuscript is highly appreciated.

Sincerely yours,

Cheng-Di Chiu MD, PhD Associate Professor, School of Medicine,

China Medical University and Hospital, Taichung, Taiwan

Telephone: +886-4- 22052121#5036; +886-928829256

Fax:	+886-4- 22053764

Email:	[cdchiu4046@gmail.com](mailto:cdchiu4046@gmail.com)

**Response to reviewers**

**Reviewer #1:**

 This study was designed to clarify the efficacy of the key lesion decompression for lumbar spinal stenosis at multiple segments in image studies using a minimally invasive percutaneous endoscopic technique (sEndo-ULBD). The key lesion was decided by history taking, physical examination and the consistency between symptoms and images. After the authors evaluated the radiographic and patient-reported clinical outcomes pre- and post-operatively in nine older patients, they concluded that key lesion sEndo-ULBD was appropriate, safe and effective treatment for degenerative LSS.
Major comment:
The authors should be commended for their efforts on this article, which required substantial effort. However, I am concerned regarding the following issues.
1. The reviewer also think that key lesion decompression using a minimally invasive surgical technique is true minimally invasive treatment. The main purpose of the current study might be to clarify the efficacy of the key lesion decompression for lumbar spinal stenosis at multiple segments in image studies. If so, a large number of patients with long-term follow-up are needed. Remaining stenotic lesions may cause symptoms 1, 2 or 5 years after index surgery even in octogenarian patients. 6-month follow-up is too short and insufficient. Did any patients, who is included or not included in this series, need to undergo additional surgery for remaining stenotic lesions during longer follow-up period?

Response:
We appreciate the reviewer's valuable comments.
We acknowledge that a 6-month follow-up may not be sufficient for a more comprehensive analysis. Our data collection commenced from January 2021 to July 2022. The submission period of our work allowed us to extend the follow-up duration for our patients. We continued collecting data until December 2023, enabling us to include outcomes for 6 patients who were followed up for 1 year and 4 patients for 1.5 years. (Table 1) However, the total number of patients did not increase, as we maintained a minimum follow-up period of 6 months. In the case of octogenarian patients, regular follow-up posed challenges, likely due to their complex medical issues and the potential need for assistance from younger companions for hospital visits.
As far as our records show, up to the last documented follow-up for our patient series, none required additional surgery for residual stenotic lesions. We add this statement in the “Discussion” paragraph (Lines 281-282)

2. Key lesion decompression may be sometimes challenging. Some older patients with lumbar spinal stenosis at multiple segments may have difficulty in choosing key lesion (selective) decompression to avoid additional surgery in the future after index surgery. How many patients with multi-segment stenosis agreed or disagreed with undergoing key lesion decompression?

Response:
We thank the reviewer for their significant remarks.
We acknowledge the shortfall in providing an exact count of all patients and add this statement to the part of the study limitation. (Lines 294-296) Traditionally, in Taiwan, surgery for octogenarian patients is often not considered a viable option. This perception stems from both patients and their families, who commonly view spine surgery as excessively risky and, culturally, as an omen of bad luck. Consequently, these octogenarian patients are more inclined to seek less invasive alternatives, such as traditional Chinese medicine or manipulation therapies.
Furthermore, long-segment spinal stenosis surgery presents even higher risks due to extended surgical times and increased blood loss, compared to key lesion endoscopic surgery. Based on our experience, it is rare for either octogenarian patients or their families to consent to long-segment spinal surgery. These factors collectively contribute to the limited number of cases in our series.

Specific comment:
1. Page 2, Line 53 (in the Conclusion section of Abstract)
The authors described that the long-term follow-up did not find any significant progression in spinal curvature or instability. The reviewer thinks that the term “long-term” is not appropriate. 6-month should be short-term.

Response:
Thank you for the reviewer's kind reminder.
We have addressed the previously misleading term and revised "long-term" to "With an average of one year’s follow-up" (Line 53-54).

**Reviewer #2:**

This paper was about”Effectiveness of percutaneous key lesion endoscopic lumbar decompression for the treatment of lumbar spinal stenosis in octogenarian patients”
Now degenerative lumbar spinal stenosis (LSS) was a big problem. It affects the quality of life and limits daily activities. The sEndo-ULBD is very suitable for treating octogenarian patients with LSS .
Key lesion sEndo-ULBD was an appropriate, safe, and effective treatment for octogenarian patients suffering from degenerative LSS. sEndo-ULBD is a good choice to to aggressive fusion fixation lumbar surgery for managing degenerative LSS in octogenarian patients with functional disability.
Limit: The number of patients was too little and follow-up need more long time.

Response:
Thank you for the reviewer's advice.
We recognize that a 6-month follow-up may not adequately suffice for a more comprehensive analysis. Our data collection spanned from January 2021 to July 2022. The submission period of our work provided an opportunity to prolong the follow-up duration for our patients. We continued to gather data until December 2023, which allowed us to include outcomes for 6 patients with a 1-year follow-up and 4 patients with a 1.5-year follow-up. (Table 1) Nevertheless, the overall patient count remained unchanged, as we adhered to a minimum follow-up period of 6 months. Regular follow-ups for octogenarian patients were challenging, often due to their intricate medical conditions and the likely necessity for younger companions to assist with hospital visits.
Our next step is to recruit more octogenarian patients with multilevel spinal stenosis and to collaborate with additional spine centers. This effort is aimed not only at increasing our patient cohort but also at extending the follow-up duration. This will help us gain a clearer understanding of the effectiveness and efficiency of key-lesion endoscopic surgery.

**Reviewer #3:**

 THE MANUSCRIPT EXPLAINS IN DETAIL THE NEED FOR THE STUDY , THE BENEFITS OF THE STUDY , THE OUTCOME OF THE PROCEDURES AND NEED FOR PARADIGM SHIFT IN THE MANAGEMENT OF THESE CONDITIONS IN THE [ELDERLY. IT](http://ELDERLY.IT) IS THUS WORTH PUBLISHING

Response: Thank you for the reviewer’s positive feedback.
